# Supplementary material for: Different Factors Influencing Postural Stability during Transcutaneous Electrical Stimulation of the Cervical Spinal Cord
Source: J Funct Morphol Kinesiol. 2024 Aug 22;9(3):142. doi: 10.3390/jfmk9030142 (PMC11417861; doi:10.3390/jfmk9030142)
Supplement: Supplementary file 1 [file jfmk-09-00142-s001.zip › Supplementary Materials_spectral parameters.pdf]

Table S5. *Spectral parameters with subthreshold and suprathreshold 5 Hz transcutaneous electrical stimulation of the spinal cord (tSCS)*

**Indicators**	**EOHS + tSCS**		**ECHS + tSCS**		**EOSS + tSCS**		**ECSS + tSCS**	
	**Before tSCS**	**2nd minute of tSCS**	**Before tSCS**	**2nd minute of tSCS**	**Before tSCS**	**2nd minute of tSCS**	**Before tSCS**	**2nd minute of tSCS**
subthreshold tSCS								
Pw1(S)0.02-0.2, %	34,17±7,87	33,81±10,73	37,21±6,87	31,24±8,91	33,22±8,23	38,25±10,08	25,52±6,10	25,95±6,55
Pw2(S)0.1-1, %	57,54±4,77	58,96±4,68	60,89±5,17	57,66±6,19	61,02±7,33	55,49±8,82	60,58±4,70	61,61±5,52
Pw3(S)0.5-2, %	31,19±4,06	31,57±9,30	27,44±5,21	34,01±5,79	31,39±4,99	29,04±7,91	38,77±6,85	37,54±6,74
Pw4(S)2-5, %	8,65±1,72	8,92±3,53	6,92±1,80	8,76±0,97	8,76±2,01	8,71±2,44	11,26±2,69	10,21±2,98
Pw1(F)0.02-0.2, %	37,42±4,22	38,49±11,17	36,50±12,73	30,62±6,17	30,08±5,99	35,39±3,72	23,95±8,98	25,84±8,46
Pw2(F)0.1-1, %	61,79±2,75	61,09±6,29	62,66±5,37	63,57±3,27	61,99±5,75	63,19±6,20	63,19±5,97	64,96±3,42
Pw3(F)0.5-2, %	26,67±4,76	26,11±7,40	25,71±7,49	30,50±6,65	33,05±4,36	28,43±5,45	39,03±4,43	33,38±4,88
Pw4(F)2-5, %	6,56±1,12	6,42±1,82	7,11±2,49	7,73±1,78	7,92±2,24	7,09±1,88	8,67±1,78	8,08±2,02
suprathreshold tSCS								
Pw1(S)0.02-0.2, %	35,59±10,41	37,05±7,16	32,07±8,79	31,75±6,78	37,67±2,12	30,55±2,05	23,75±5,39	25,14±11,03
Pw2(S)0.1-1, %	56,55±7,38	56,29±7,90	61,12±6,99	61,39±7,79	57,44±4,46	60,31±7,67	58,05±6,52	57,62±6,67
Pw3(S)0.5-2, %	31,71±9,03	30,83±5,07	31,79±3,57	30,47±2,19	31,88±3,03	32,39±2,77	37,54±8,17	38,81±9,91
Pw4(S)2-5, %	8,72±0,95	9,03±1,52	7,55±1,12	7,99±0,82	8,43±1,78	10,16±2,27	10,98±4,05	11,89±5,38
Pw1(F)0.02-0.2, %	28,97±5,78	30,57±5,76	30,74±9,22	28,04±9,75	29,34±5,92	32,90±3,63	23,59±6,23	30,81±7,97
Pw2(F)0.1-1, %	60,65±4,77	59,55±6,79	61,12±4,65	64,30±4,74	61,62±4,05	56,26±6,45	64,09±3,16	59,13±4,30
Pw3(F)0.5-2, %	34,82±7,87	32,55±4,01	31,76±10,57	31,70±4,69	35,67±7,39	34,74±2,27	37,25±4,92	33,10±4,67
Pw4(F)2-5, %	8,76±2,23	8,29±2,16	7,81±1,47	7,67±3,71	8,93±2,01	9,26±1,95	8,67±0,97	9,39±2,11

EOHS – standing on a hard surface with open eyes
ECHS – standing on a hard surface with closed eyes
EOSS – standing on a soft surface with open eyes
ECSS – standing on a soft surface with closed eyes

Table S6. *Spectral parameters with subthreshold and suprathreshold 30 Hz transcutaneous electrical stimulation of the spinal cord (tSCS)*

**Indicators**	**EOHS + tSCS**		**ECHS + tSCS**		**EOSS + tSCS**		**EC SS + tSCS**	
	**Before tSCS**	**2nd minute of tSCS**	**Before tSCS**	**2nd minute of tSCS**	**Before tSCS**	**2nd minute of tSCS**	**Before tSCS**	**2nd minute of tSCS**
subthreshold tSCS								
Pw1(S)0.02-0.2, %	37,57±5,24	34,37±6,99	28,37±10,01	29,50±10,50	30,62±6,74	32,67±6,68	24,49±9,64	22,36±9,37
Pw2(S)0.1-1, %	54,78±4,84	58,54±4,22	56,27±5,04	55,88±7,48	58,84±4,79	59,38±5,13	54,08±8,47	57,39±6,63
Pw3(S)0.5-2, %	30,49±7,54	31,75±5,78	38,32±9,40	35,37±10,76	34,58±4,98	33,35±6,03	37,89±6,57	38,62±7,04
Pw4(S)2-5, %	7,78±1,27	7,73±1,53	9,89±3,83	9,65±4,36	9,31±2,36	9,01±2,36	13,18±5,79	13,19±6,25
Pw1(F)0.02-0.2, %	30,88±8,16	31,77±6,80	31,79±4,94	32,40±9,43	30,95±7,42	31,93±7,84	21,63±4,73	24,68±6,68
Pw2(F)0.1-1, %	59,30±6,59	61,19±5,79	60,24±4,67	61,23±5,75	59,09±5,22	57,07±5,82	59,95±4,76	58,14±6,53
Pw3(F)0.5-2, %	32,45±5,04	32,07±4,14	33,89±5,31	29,69±6,82	34,62±7,08	33,54±5,00	41,62±6,44	37,88±7,01
Pw4(F)2-5, %	7,39±1,56	7,47±1,25	8,08±2,21	7,29±1,58	8,75±1,69	8,53±1,07	11,17±3,14	10,64±2,78
suprathreshold tSCS								
Pw1(S)0.02-0.2, %	34,59±8,19	32,88±7,92	28,62±10,42	31,43±7,54	31,88±7,07	32,85±7,39	23,90±9,66	23,05±5,02
Pw2(S)0.1-1, %	57,04±5,31	53,39±6,55	57,20±8,16	57,21±4,25	58,52±5,56	57,47±5,28	56,27±9,07	57,90±6,92
Pw3(S)0.5-2, %	32,94±5,30	33,10±5,64	37,83±10,51	33,87± 6,98	36,39± 5,93	33,81±5,33	38,53±6,45	38,95±6,89
Pw4(S)2-5, %	8,77±1,84	10,06±3,18	9,82±3,19	9,32±2,22	9,16±2,07	8,96±2,55	13,35±9,44	12,56±5,39
Pw1(F)0.02-0.2, %	27,61±3,51	34,26±6,80	26,95±5,07	29,00±6,55	30,10±5,54	31,59±6,25	23,38±3,53	23,92±5,44
Pw2(F)0.1-1, %	60,67±6,26	59,29±6,37	61,43±4,94	61,99±5,84	59,61±6,69	56,67±6,29	59,26±4,81	59,09±5,98
Pw3(F)0.5-2, %	34,39±3,55	31,41±7,13	32,88±4,77	32,04±5,35	35,12±3,69	35,88±6,06	38,66±3,64	38,06±6,98
Pw4(F)2-5, %	8,35±2,36	7,49±1,42	8,74±1,76	7,96±1,80	8,82±1,47	8,65±1,75	11,06±2,26	10,61±3,39

EOHS – standing on a hard surface with open eyes
ECHS – standing on a hard surface with closed eyes
EOSS – standing on a soft surface with open eyes
ECSS – standing on a soft surface with closed eyes
