# Supplementary material for: Different Factors Influencing Postural Stability during Transcutaneous Electrical Stimulation of the Cervical Spinal Cord
Source: J Funct Morphol Kinesiol. 2024 Aug 22;9(3):142. doi: 10.3390/jfmk9030142 (PMC11417861; doi:10.3390/jfmk9030142)
Supplement: Supplementary file 1 [file jfmk-09-00142-s001.zip › Supplementary Materials_Stabilographic parameters.pdf]

Table S3. *Stabilographic parameters with subthreshold and suprathreshold 5 Hz transcutaneous electrical stimulation of the spinal cord (tSCS)*

**Indicators**	**EOHS + tSCS**		**ECHS + tSCS**		**EOSS + tSCS**		**EC SS + tSCS**	
	**Before tSCS**	**2nd minute of tSCS**	**Before tSCS**	**2nd minute of tSCS**	**Before tSCS**	**2nd minute of tSCS**	**Before tSCS**	**2nd minute of tSCS**
subthreshold tSCS								
EllS – area of ellipse (static kinesiogram), mm^2	205,5±191,3	160,9±122,4	336±297,3	222,8±156,9	638,3±381,2*	658,9±516,6	1522,9± 907,9*#	1386,7±839,5
Qy – root mean square deviation in sagittal (Y) plane, mm	4,27±2,06	3,34±1,56	5,60±2,82	3,97±1,64	7,29±2,69*	6,55±2,93	10,31±3,21*#	9,78±4,11
Qx – root mean square deviation in frontal (X) plane, mm	2,99±1,76	3,01±1,44	4,14±2,22	3,65±1,66	5,74±2,09*	6,28±2,31	9,59±3,01*#	9,20±2,57
ALV – average linear velocity, mm/s	7,75±3,76	7,56±3,46	10,18±4,45#	10,27±5,72	14,70±4,79*	12,74±2,49	33,73±11,94*#	29,33±8,46@
AAV – average angular velocity, deg/s	19,26±3,07	20,79±1,73	17,86±2,49	19,90±3,20	15,64±2,27*	16,56±3,40	13,83±1,20*	14,37±0,90
suprathreshold tSCS								
EllS – area of ellipse (static kinesiogram), mm^2	206,6±197,5	147,4±113,2	319,8±183,7	190,5±130,8	576,5±322,9	398,3±184,5	1143,2±813,5*	1198,1±838,2
Qy – root mean square deviation in sagittal (Y) plane, mm	4,60±3,05	3,37±1,43	5,86±2,64	4,34±2,44	7,22±2,97	5,17±1,83	9,07±3,98	9,97±5,17
Qx – root mean square deviation in frontal (X) plane, mm	2,73±1,33	2,87±1,45	3,79±0,99	3,16±0,75	5,28±1,04*	5,53±1,45	8,12±2,10*#	8,29±2,69
ALV – average linear velocity, mm/s	8,10±3,36	7,33±2,53	10,69±3,41	8,47±2,31	14,91±2,82*	13,25±2,57	28,57±8,24*#	26,78±4,18
AAV – average angular velocity, deg/s	20,82±4,40	22,06±4,51	17,56±1,67	19,16±2,18	15,28±1,56*	17,92±2,32$	14,52±2,29	15,04±1,09$

EOHS – standing on a hard surface with open eyes
ECHS – standing on a hard surface with closed eyes
EOSS – standing on a soft surface with open eyes
ECSS – standing on a soft surface with closed eyes

* – differences between without and with soft surface
# – differences between the EO and EC
$ – differences between the subthreshold and suprathreshold tSCS
@ – differences between the Before tSCS and 2nd minute of tSCS
The significance level was set at p <0.05.

Table S4. *Stabilographic parameters with subthreshold and suprathreshold 30 Hz transcutaneous electrical stimulation of the spinal cord (tSCS)*

**Indicators**	**EOHS + tSCS**		**ECHS + tSCS**		**EOSS + tSCS**		**EC SS + tSCS**	
	**Before tSCS**	**2nd minute of tSCS**	**Before tSCS**	**2nd minute of tSCS**	**Before tSCS**	**2nd minute of tSCS**	**Before tSCS**	**2nd minute of tSCS**
subthreshold tSCS								
EllS – area of ellipse (static kinesiogram), mm^2	114,2±71,74	69,14±41,31 @	138,7±73,28	117,1±68,32	334,9±162,3*	304,1±183,2	872,5±134,9 *#	895,6±305,4
Qy – root mean square deviation in sagittal (Y) plane, mm	3,37±1,15	2,49±0,79@	3,80±1,03	3,37±1,13#	4,86±1,24*	4,88±1,47	8,63±1,07*#	8,45±1,89
Qx – root mean square deviation in frontal (X) plane, mm	2,31±0,82	1,86±0,61@	2,48±0,84	2,31±0,84	4,57±1,35*	4,05±1,32	7,06±0,89*#	7,47±1,46
ALV – average linear velocity, mm/s	5,92±1,80	5,45±1,80	9,02±4,22#	8,05±3,96	12,58±3,10*	11,59±3,36@	33,45±9,98* #	30,58±7,19
AAV – average angular velocity, deg/s	19,51±4,07	20,56±5,28	17,47±3,34	17,44±4,15	15,53±2,17*	15,83±2,15	15,31±3,64	15,29±3,18
suprathreshold tSCS								
EllS – area of ellipse (static kinesiogram), mm^2	109,4±64,01	102,5±84,38	154,0±93,93	99,87±56,52 @	320,1±169,5*	262,3±102,1	906,7±233,8 *#	878,3±351,4
Qy – root mean square	3,11±1,04	2,82±1,16	3,91±2,09	2,87±0,92$	5,15±1,52*	4,66±1,57	8,89±1,84*#	8,72±1,62

deviation in sagittal (Y) plane, mm								
Qx – root mean square deviation in frontal (X) plane, mm	2,38±0,72	2,28±1,18	2,72±0,74	2,33±0,74	4,15±1,12*	3,92±0,79	7,27±1,15*#	6,91±1,33
ALV – average linear velocity, mm/s	6,65±2,66	6,15±2,85	9,18±3,66	7,55±3,37@	12,49±3,69*	11,63±2,88	31,58±8,93* #	29,39±7,41
AAV – average angular velocity, deg/s	19,22±4,66	19,71±4,45	17,24±3,39	18,08±3,43	15,41±2,67*	16,02±2,96	14,57±2,79	14,64±3,06

EOHS – standing on a hard surface with open eyes
ECHS – standing on a hard surface with closed eyes
EOSS – standing on a soft surface with open eyes
ECSS – standing on a soft surface with closed eyes

* – differences between without and with soft surface
# – differences between the EO and EC
$ – differences between the subthreshold and suprathreshold tSCS
@ – differences between the Before tSCS and 2nd minute of tSCS
The significance level was set at $p < 0.05$.
