# Supplementary material for: Different Factors Influencing Postural Stability during Transcutaneous Electrical Stimulation of the Cervical Spinal Cord
Source: J Funct Morphol Kinesiol. 2024 Aug 22;9(3):142. doi: 10.3390/jfmk9030142 (PMC11417861; doi:10.3390/jfmk9030142)
Supplement: Supplementary file 1 [file jfmk-09-00142-s001.zip › Supplementary Materials_Table1,2.pdf]

Table S1. Anthropometric data of the participants

№	Participants	Sex	Height, cm	Weight, kg	Age, years
1	p1 (BL)	female	157	50	25
2	p2 (PA)	female	159	57	20
3	p3 (MiA)	female	156	48	19
4	p4 (KS)	female	158	55	19
5	p5 (DS)	female	158	46	19
6	p6 (SA)	female	161	51	18
7	p7 (AI)	male	178	100	20
8	p8 (TR)	male	185	70	24
9	p9 (OV)	male	186	90	19
10	p10 (ShL)	female	157	65	19
11	p11 (ShV)	female	159	45	27
12	p12 (ZhA)	female	159	46	25
13	p13 (PA2)	female	156	48	22
14	p14 (NM)	female	163	59	21
**Mean**			**163.71**	**59,29**	**21,21**
**Standard dev.**			**10.75**	**16,96**	**2,88**

Table S2. Analyzed center of pressure (CoP) parameters.

Parameter	Definition	Formula
Confidence ellipse area, ELLS, mm^2	The main part of the area occupied by the CoP without socalled loops and accidental outliers	$ELLS = 2ln\frac{1}{1-\beta}\sqrt{D(X)D(Y) - Cov(X,Y)^2}$<br>β – probability that the point of the statokinesiogram hits into the ellipse (β = 0.9).<br>D(X), D(Y) – corresponding component dispersion
RMSD along the frontal axis, Qx, mm	Root mean square deviation of the CoP position along the frontal axis	$Q_x = \sqrt{\frac{1}{N-1}\sum_{i=1}^{N}\left(X_i - X_{cp}\right)^2}$<br>Xi– CoP coordinates in time<br>N – number of counts
RMSD along the sagittal axis, Qy, mm	Root mean square deviation of the CoP position along the sagittal axis	$Q_y = \sqrt{\frac{1}{N-1}\sum_{i=1}^{N}\left(Y_i - Y_{cp}\right)^2}$<br>Yi – CoP coordinates in time<br>N – number of counts
ALV, mm/s	The average linear velocity of the CoP movement, represented by the ratio of the length of the path of movement of the CoP to the duration of the test	$V_{cp} = \frac{1}{N}\sum_{i=1}^{N}\frac{V_i}{T}$<br>$V_i$ – instantaneous value of the velocity vector<br>T – experimental time
AAV, deg/s	The average angular velocity of the CoP movement – the average amplitude of the velocity of the participant's CoP during the duration of the examination	$\Omega_{cp} = \frac{1}{N}\sum_{i=1}^{N}\frac{\Delta\varphi_i}{T_Д}$<br>$\Delta\varphi_i$ – current change in the velocity vector angle<br>$T_Д$ – sampling time<br>N – number of velocity vectors
